# Hormone-receptor expression and survival patterns in operated cases of female invasive ductal breast carcinoma in Kerala: a retrospective cohort study

**DOI:** 10.1186/s12957-015-0582-x

**Published:** 2015-04-23

**Authors:** Ajith Vettuparambil, Ravindran Chirukandath, Terence B Culas, Sajna Mathumkunnath Vijayan, Gautham Rajan, Sathidevi Vadakkepura Kuttappan

**Affiliations:** Department of General Surgery, Government Medical College, Thrissur, Kerala 680596 India; Department of Community Medicine, Government Medical College, Thrissur, Kerala 680596 India; Government Medical College, Thrissur, Kerala 680596 India; Department of Anatomy (Epidemiology), Government Medical College, Thrissur, Kerala 680596 India

**Keywords:** Hormone receptor, Survival, Breast cancer, Kerala, India

## Abstract

**Background:**

Though breast cancer is the most common cancer among women in Kerala, India, epidemiological data on breast cancer in the state is largely lacking. The objectives of this study were to analyze the survival pattern of female breast carcinoma in this region of the country and to compare the differences in survival with different hormone-receptor expressions.

**Methods:**

One hundred eighty-nine female breast cancer patients who were operated between 1 August 2008 and 3 July 2009 were followed up over telephone to obtain data on five-year survival. Grade, stage of the disease, and hormone-receptor (HR) status were obtained from treatment records. Logistic regression and the Kaplan-Meier survival analysis were used for statistical analysis.

**Results:**

The mean age of the study population was 49.07 (SD, 10.35) years. A majority of the patients had estrogen receptor (ER)+/progesterone receptor (PR) + tumors (*n* = 103, 54.5%), followed by 72 (38.1%) ER−/PR−, 10 (5.3%) ER−/PR+, and 4 (2.1%) ER+/PR−. Stage of the disease, axillary nodal status, and hormone-receptor status showed statistically significant association with overall survival in breast cancer. Overall survival rate at the end of 5 years was 71.4%. Mortality was found to be highest for the ER − PR − group (47.2%).

**Conclusions:**

Women in Kerala are diagnosed with breast carcinoma at a relatively younger age, yet the overall five-year survival for the disease is low when compared to developed nations. It is imperative that comprehensive breast cancer screening and treatment strategies be developed to enable earlier diagnosis and improve the survival of breast cancer in the state.

## Background

Breast cancer is the most common cancer among women in many regions of India [1-3]. It is highly heterogeneous, with a wide range of biological, pathological, and clinical characteristics [4]. Among these, hormone receptors (estrogen receptor (ER), progesterone receptor (PR)) greatly influence clinical outcome, and their role as a prognostic and therapeutic tool is widely accepted [5,6]. Though the incidence of breast cancer in Kerala has been steadily mounting, data on epidemiology and survival of breast cancer in the state is scarce.

The objectives of this study were to analyze the survival pattern of female breast carcinoma in this region of the country and to compare the differences in survival with different hormone-receptor expressions.

## Methods

A retrospective cohort study was conducted on 189 female patients who were diagnosed with invasive ductal carcinoma and had undergone surgery between 1 August 2008 and 31 July 2009 in the Department of General Surgery, Government Medical College, Thrissur. The required information was collected from the treatment records of the patients maintained at the Medical Records Library. All data including age, menopausal status, and pathological characteristics [grade (modified Bloom-Richardson grade), stage of the disease (AJCC), tumor size, and axillary nodal status] were recorded. Patients whose hormone-receptor status was not analyzed were excluded from the study. Patients who had either ER or PR positive tumors were treated with hormonal therapy. Overall survival was calculated in months, either from the date of diagnosis or from the date of surgery up to 31 December, 2013. Five-year survival at the end of the study period was obtained from follow-up records for those who were on regular follow-up and was confirmed by contacting the patients or their relatives over telephone. Those who had been lost to follow-up were also traced over telephone.

All post-mastectomy specimens were evaluated for hormone-receptor expression (ER and PR) by immunohistochemistry. In accordance with the American Society of Clinical Oncology (ASCO) guidelines [7], which were being followed when the assays were done, specimens in which more than 10% of the tumor-nuclei stained positive were reported hormone-receptor positive. ER status was determined using the BioGenex monoclonal mouse IgG (Clone 1D5) (BioGenex, USA), and PR status was determined using BioGenex monoclonal mouse IgG (Clone 1A6) (BioGenex, USA). Antigen retrieval was done using the BioGenex EZ-Retriever system (BioGenex, USA). Based on hormone-receptor expression, patients were grouped into four categories, that is, ER + PR+ (ER positive PR positive), ER + PR−(ER positive PR negative), ER − PR+ (ER negative PR positive), and ER − PR−(ER negative PR negative).

Pathologic variables were compared using chi-square test. Logistic regression analysis was carried out to assess the independent association between different variables and survival. The Kaplan-Meier method was used to estimate the overall survival.

## Results

One hundred eighty-nine female patients with invasive ductal breast cancer were included in the study. The age of the patients ranged from 27 to 80 with a median of 49.07 ± 10 years. ER expression was seen in 107 patents (56.6%) and PR expression in 113 patients (59.8%).

### General characteristics

The general characteristics of the study group are shown in Table 1. Out of the 189 patients, 111 (58.7%) were pre-menopausal. One hundred twenty-seven patients (67.2%) were between 41 and 60 years of age. Distribution of patients according to their age and hormone-receptor expression is shown in Table 2. A majority (*n* = 127, 67.2%) had stage 2 breast cancer at presentation. One hundred four patients (55%) had grade 2 tumor, and 120 (63.2%) had tumor size between 2 and 5 cm.

**Table 1:** **General characteristics of the study population**

**Variable**		***N*** **(number)**	**%**
Menopausal			
	Pre-menopausal	111	58.73
	Post-menopausal	78	41.27
Grade	I	13	6.8
	II	104	55
	III	72	38.1
Stage	I	11	5.8
	II	127	67.2
	III	51	27
Tumor size			
	T1	14	7.4
	T2	120	63.2
	T3	34	17.9
	T4	21	11.1
ER status			
	ER+	107	56.61
	ER−	82	43.39
PR status			
	PR+	113	60
	PR−	76	40
Hormone-receptor status			
	ER + PR+	103	54.5
	ER + PR−	4	2.1
	ER − PR+	10	5.3
	ER − PR−	72	38.1

**Table 2:** **Distribution of patients according to their age and HR expression and their outcome**

**Age group**	**Total**	**ER + PR+**	**ER + PR−**	**ER − PR+**	**ER − PR−**	**Survival**
	***N***	***N*** **(%)**	***N*** **(%)**	***N*** **(%)**	***N*** **(%)**	***N*** **(%)**
21 to 30	5	1 (20)	0	0	4 (80)	1 (20)
31 to 40	36	23 (63.8)	1 (2.78)	3 (8.33)	9 (25)	27 (72.97)
41 to 50	79	46 (58.23)	0	2 (2.53)	31 (39.24)	59 (75.64)
51 to 60	48	20 (41.67)	3 (6.25)	5 (10.42)	20 (41.66)	35 (72.91)
61 to 70	16	10 (62.5)	0	0	6 (37.5)	11 (68.75)
71 to 80	5	3 (60)	0	0	2 (40)	2 (40)
Total	189	103	4	10	72	

### Hormone-receptor expression and outcome

**Figure 1:**
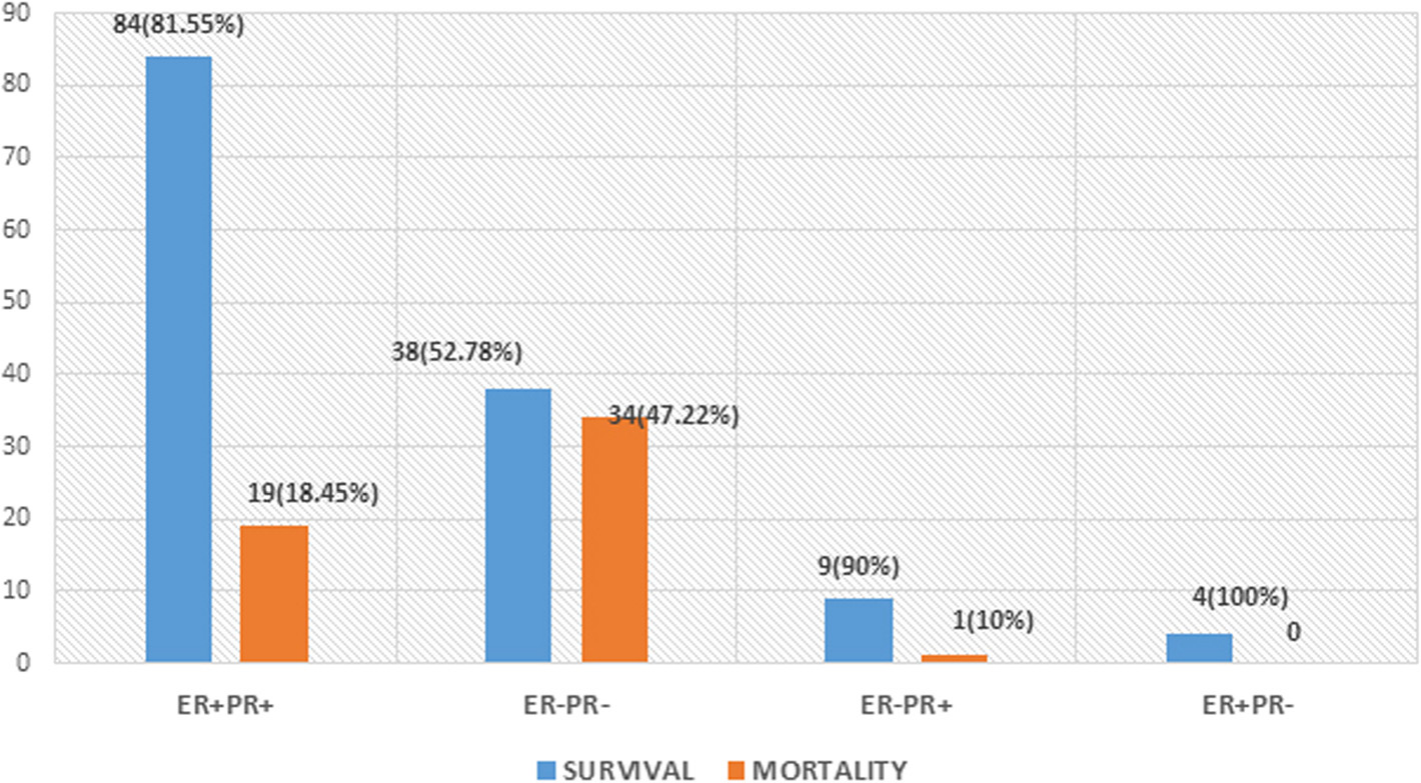
Pattern of hormone-receptor expression and survival.

**Figure 2:**
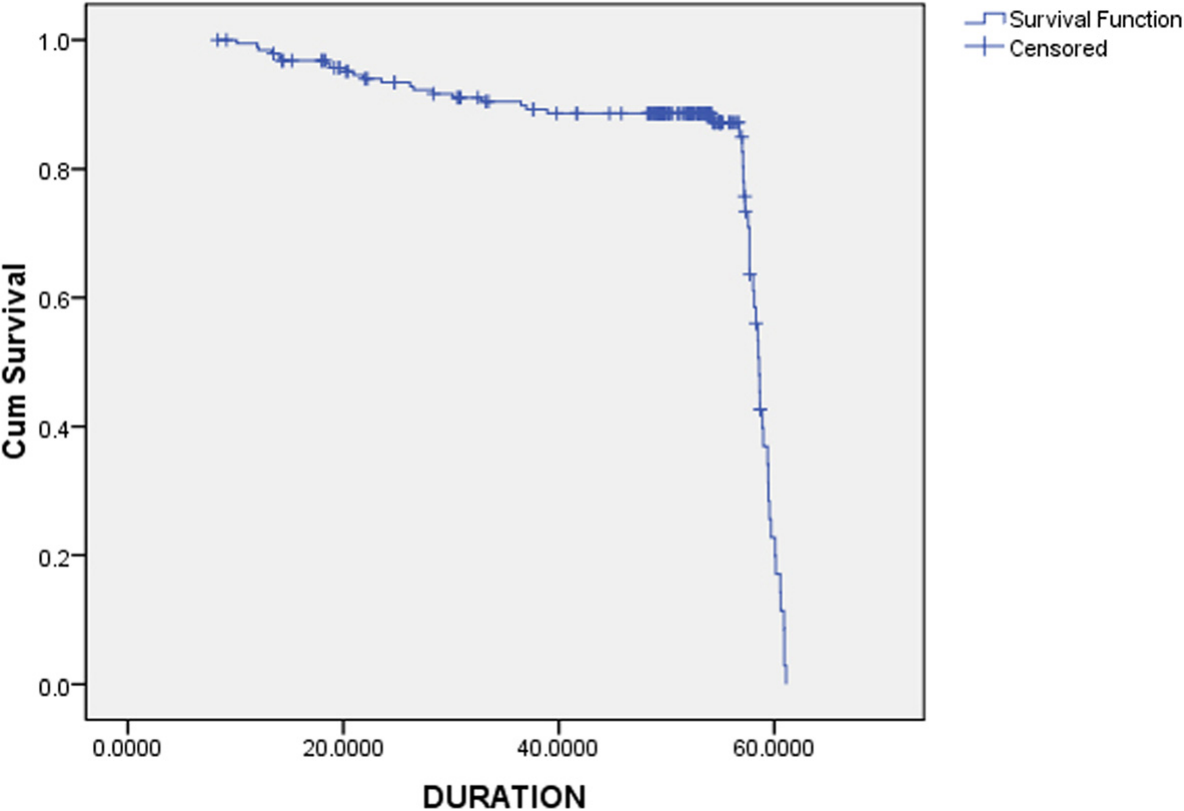
Kaplan-Meier Curve for overall survival.

**Figure 3:**
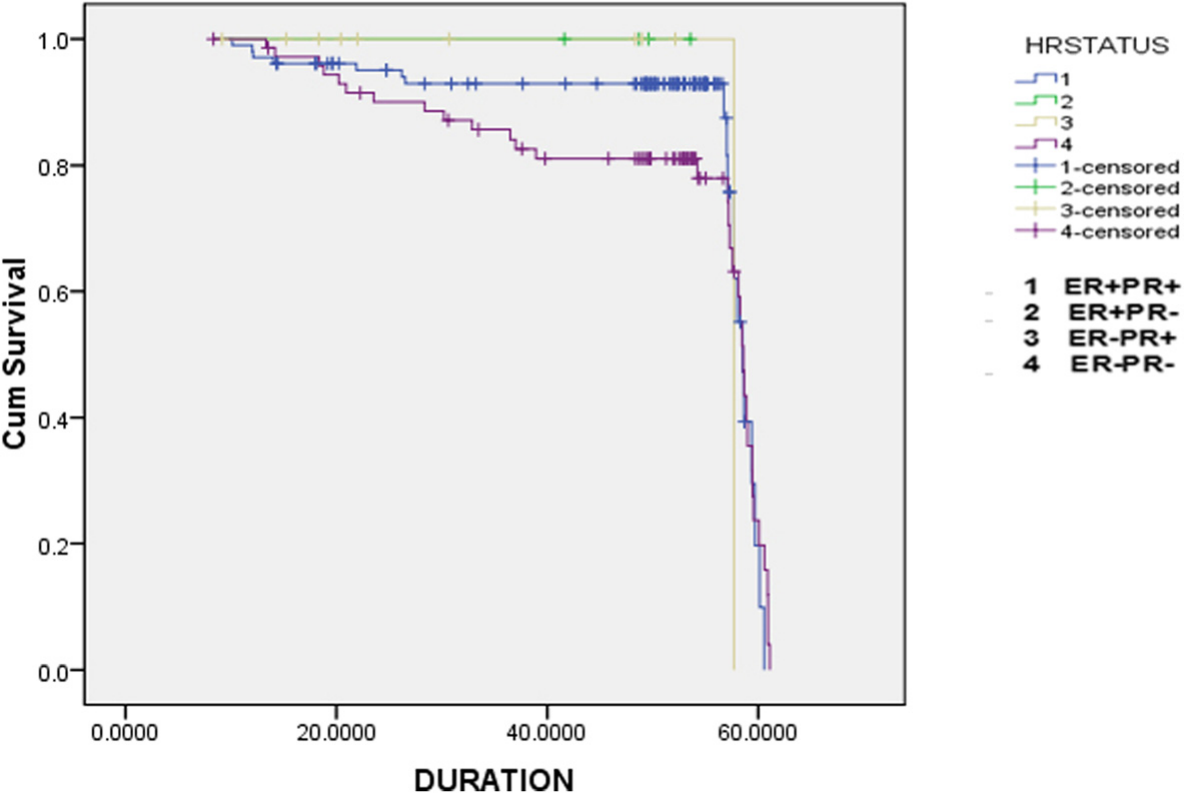
Kaplan-Meier Curve for survival for different hormone-receptor expression.

In this breast cancer cohort, 103 patients (54.5%) were ER + PR+, 72 patients (38.1%) were ER − PR−, 10 patients (5.3%) were ER − PR+, and 4 patients (2.1%) ER + PR−. Mean follow-up time was 52 months. 71.4% of the patients (*n* = 135) were found to have survived to five years from the time of diagnosis. Mortality was found to be highest for the ER − PR − group (*n* = 38, 47.2%). Data regarding the four types of hormone-receptor expression and the outcomes of patents that belonged to each group is shown in Figure 1. Among the different age groups, patients aged 50 years or younger were found to have a higher mortality (Table 2). The Kaplan-Meier survival curve was plotted for the different types of hormone-receptor expression and for overall survival (Figures 2 and 3).

Tables 3 and 4 show the association between different variables and survival in breast cancer. Stage of the disease (adjusted risk ratio (RR) 1.274), nodal status (adjusted RR 1.055), and hormone-receptor status (adjusted RR 2.859) showed statistically significant association with overall survival.

**Table 3:** **Association of different variables to survival of breast cancer (univariate analysis)**

**Pathological variables and other factors**	***P*** **value**
Age	0.083
Menopausal status	0.53
Tumor size	0.164
Tumor grade	0.084
Stage	<0.0001
Nodal status	0.0001
Hormone-receptor status	<0.0001
Estrogen receptor	0.000
Progesterone receptor	0.573

*P* value <0.05 significant.

**Table 4:** **Association of pathological variables and survival in invasive female ductal carcinoma (multilogistic regression analysis)**

	**Adjusted RR**	**95% CI for adjusted RR**	
		**Lower**	**Upper**
Tumor grade	1.451	0.73	2.884
Axillary nodal status	1.393	1.055	2.571
Stage of the disease	1.958	1.274	3.007
HR status	2.859	0.962	10.733

RR - risk ratio. Cox-Snell *R* ^*2*^ 0.271.

## Discussion

Breast cancer is the most common cancer among urban women and second most common among rural women in India [1]. Breast cancer is a heterogeneous disease that can be classified into several subtypes on the basis of various clinical and pathological features [8]. Hormone-receptor (ER and PR) status is now routinely determined for all patients to assess the possibility of providing them specific adjuvant hormone therapy [6].

The median age of the study group was 49.07 ± 10.35 years, which is very low compared to the USA, where the median age at diagnosis of breast cancer is 61 years [9-11]. This considerable difference can likely be due to the genetic, racial, and socioeconomic differences between the two populations.

Data from the study showed that 56.6% patients were ER+ and 54.5% patients were ER + PR+. This is low when compared to data from the USA, where the overall ER positivity is reported to be 77% [10]. The findings are consistent with earlier studies from India [12-15]. We also have a higher proportion of ER − PR − tumors compared to the West. It has been noted that ER and PR expression in the study subjects increase with age. This is in concordance with earlier studies [16,17]. A greater number of ER − PR − tumors were seen in patients younger than 50 years of age.

Population-based studies on breast cancer in Bangalore [18] and Chennai [19] showed five-year survival rates of 42.3% and 48%, respectively. The five-year survival rate of the current study cohort was much higher, at 71.43%. The differences in the survival rate can be explained by differences in the study settings. The USA has an average five-year survival rate of 89.2% for patients diagnosed with breast cancer [10]. The overall survival rate is still low compared to the West. Five-year survival rates of more than 80% have been reported in many studies from the West and also from a developed Asian country [20,21]. Ethnicity and race have been documented as important factors that influence the survival rate [22]. Along with these factors, well-established screening programs and early detection of the disease also help improve the survival rate.

ER + PR+ breast cancer has been independently associated with decreased breast cancer mortality in many studies [4,19,23,24]. A similar survival pattern was also noted in the current cohort, with highest mortality in the ER − PR − group.

Stage of the disease, axillary nodal status, and hormone-receptor status showed statistically significant association with overall survival in breast cancer. Among these, the axillary nodal status and stage of the disease are established prognostic factors for carcinoma breast [5,16]. Along with their proven role in determining therapeutic course, hormone receptors (ER and PR) can also be used as a predictive tool for breast cancer survival.

ER − PR+ is a rare subtype of breast cancer based on hormone-receptor expression that accounts for 1% to 4% of all cases reported in literature [25]. The study cohort has a similar fraction of patients (5.3%) with ER − PR+ expression, and 90% survival has been noted in this particular group. The predictive value of PR in the absence of ER expression is controversial [26,27]. Some reports suggest that positive PR in the absence of ER has a higher response to hormone therapy, but this finding is not universal [28-30]. Unfortunately, because of the relatively small number of patients in the study group who belonged to this subset, it is difficult to comment on the role of this particular type of receptor expression on disease outcome.

## Conclusions

The overall five-year survival rate of breast cancer patients in Kerala is low compared to developed nations. The lack of an effective cancer screening program is an important reason for this. The higher proportion of hormone-receptor (HR)-negative tumors in our population may be due to delayed diagnosis along with other genetic, ethnic, and cultural variations. Since data on epidemiology and risk factors of the breast cancer in the state is inadequate, it is very crucial to further explore these aspects of the disease to develop a prevention and control strategy to improve the survival rate.

## Abbreviations

HR status: Hormone-receptor status
ER: Estrogen receptor
PR: Progesterone receptor
RR: Risk ratio
ASCO: American Society of Clinical Oncology
AJCC: American Joint Committee on Cancer
